# Intercellular Bridges in Vertebrate Gastrulation

**DOI:** 10.1371/journal.pone.0020230

**Published:** 2011-05-25

**Authors:** Luca Caneparo, Periklis Pantazis, William Dempsey, Scott E. Fraser

**Affiliations:** Beckman Institute and Division of Biology, California Institute of Technology, Pasadena, California, United States of America; Ecole Normale Supérieure de Lyon, France

## Abstract

The developing zebrafish embryo has been the subject of many studies of regional patterning, stereotypical cell movements and changes in cell shape. To better study the morphological features of cells during gastrulation, we generated mosaic embryos expressing membrane attached Dendra2 to highlight cellular boundaries. We find that intercellular bridges join a significant fraction of epiblast cells in the zebrafish embryo, reaching several cell diameters in length and spanning across different regions of the developing embryos. These intercellular bridges are distinct from the cellular protrusions previously reported as extending from hypoblast cells (1–2 cellular diameters in length) or epiblast cells (which were shorter). Most of the intercellular bridges were formed at pre-gastrula stages by the daughters of a dividing cell maintaining a membrane tether as they move apart after mitosis. These intercellular bridges persist during gastrulation and can mediate the transfer of proteins between distant cells. These findings reveal a surprising feature of the cellular landscape in zebrafish embryos and open new possibilities for cell-cell communication during gastrulation, with implications for modeling, cellular mechanics, and morphogenetic signaling.

## Introduction

**Figure 1:**
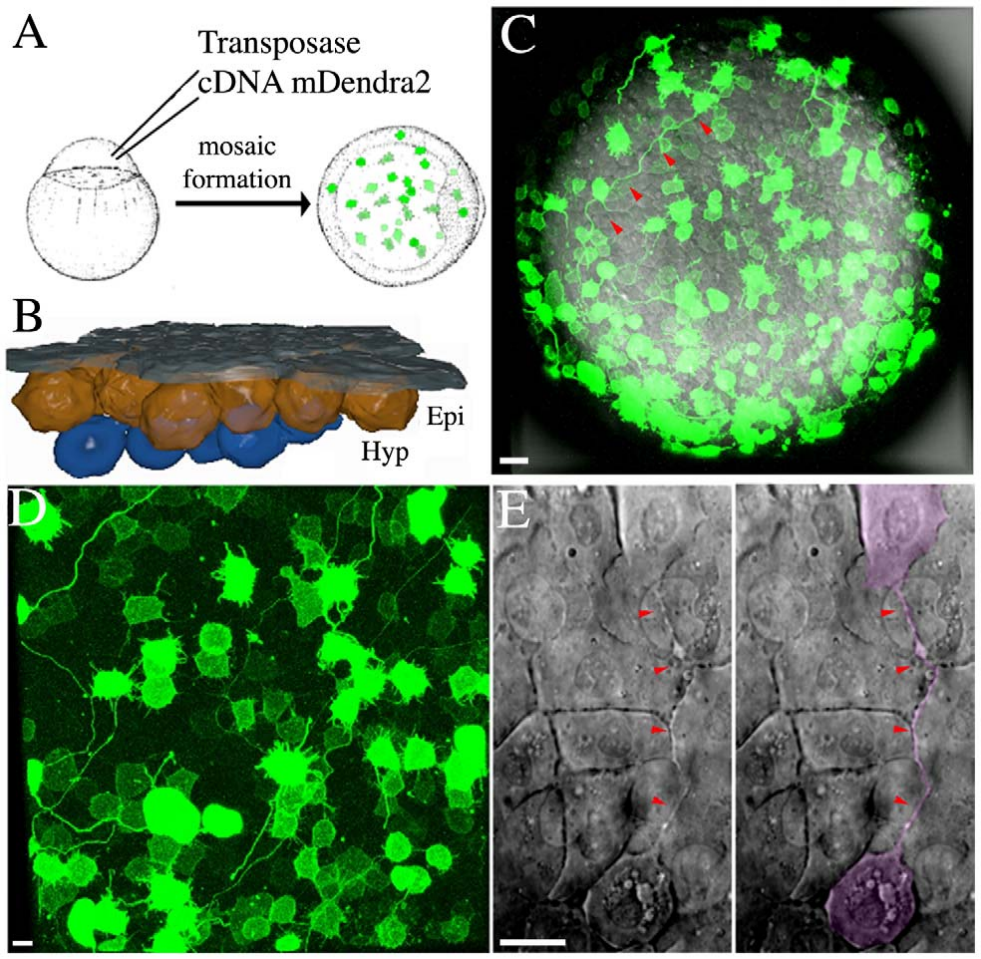
Cellular Bridge Visualisation During Gastrulation. (A) Schematic illustration of the methodology used to generate mosaic embryos with high levels of mDendra2 expression using Tol2 transposase mediated integration of an expression vector. (B) Schematic cross section of an idealized embryo at mid-gastrula stage showing the relationship between the enveloping layer (EVL; dark grey), the epiblast (Epi; orange) and the underlying migrating hypoblast (Hyp; blue). (C) Animal pole of a mosaic zebrafish embryo expressing mDendra2 at the onset of gastrulation. (D) Portion of the animal pole view, showing details of the epiblast layer with intercellular bridges depicted in D, at higher magnification. (E) DIC image of an un-injected embryo showing two epiblast cells connected by an intercellular bridge (red arrow) and highlighted in the right panel of the image in magenta. Schematics are not to scale. Scale bar (C–E): 20 µm.

At gastrula stages, zebrafish embryos have a simple organization, comprised of a few cellular layers and a relatively simple cell morphology. The enveloping layer (EVL) is the outermost part of the blastoderm, surrounding the epiblast, the hypoblast and the yolk syncytial layer (YSL), which lay on top of the yolk mass (Figure 1B) [1]. Despite having a good understanding of the overall cellular rearrangements required to define the germ layers during zebrafish gastrulation, few *in vivo* studies have focused on individual cell shapes [2], [3]. Past efforts have been mainly concentrated on the cells in the hypoblast, where blebs, filopodia and pseudopodia are notable features [1], [4], [5]. These cellular protrusions seem to lead the progressing hypoblast cells down the correct path of migration through the embryo [5]. The epiblast has instead been regarded as a single-cell thick epithelium, more homogeneous and simple [1], [2]. Compared to the hypoblast, epiblast cells are less motile with simpler morphology and with less dynamic protrusions [5], [6].

## Results and Discussion

To study cell shape in the zebrafish epiblast, we generated high cellular contrast by targeting photoconvertible protein Dendra2 [7] to the cell membrane, Tol2-mediated integration [8] of a fusion of Dendra2 to a lipid anchor recognition sequence [9], [10] (Lyn-Dendra2 referred to here as mDendra2) resulted in mosaic labeling (Figure 1A), without the need for cell transplantation or single blastomere injection, which might disrupt cellular and intercellular morphologies. This high-contrast labeling of a subset of the cells permitted confocal microscopy to obtain high-resolution images of the cell boundaries. Surprisingly, we detected the presence of cell protrusions extending up to ten cell diameters in length (Figure 1) in these mosaically labeled mDendra2 embryos during the early phases of gastrulation. Further imaging revealed that these protrusions were not filopodia; instead, they seemed to be intercellular bridges linking distant pairs of cells (see below). To rule out the possibility that these protrusions were an artifact of the expression of mDendra2, we imaged un-injected embryos at the same stage of development using differential interference contrast (DIC) light microscopy, and confirmed the presence of the previously undescribed intercellular bridges (Figure 1E). The protrusions are thin and contain little free cytoplasm, as labeling cells with rhodamine dextran or cytoplasmic Dendra2 did not highlight the protrusions (see Figure S1A and data not shown).

**Figure 2:**
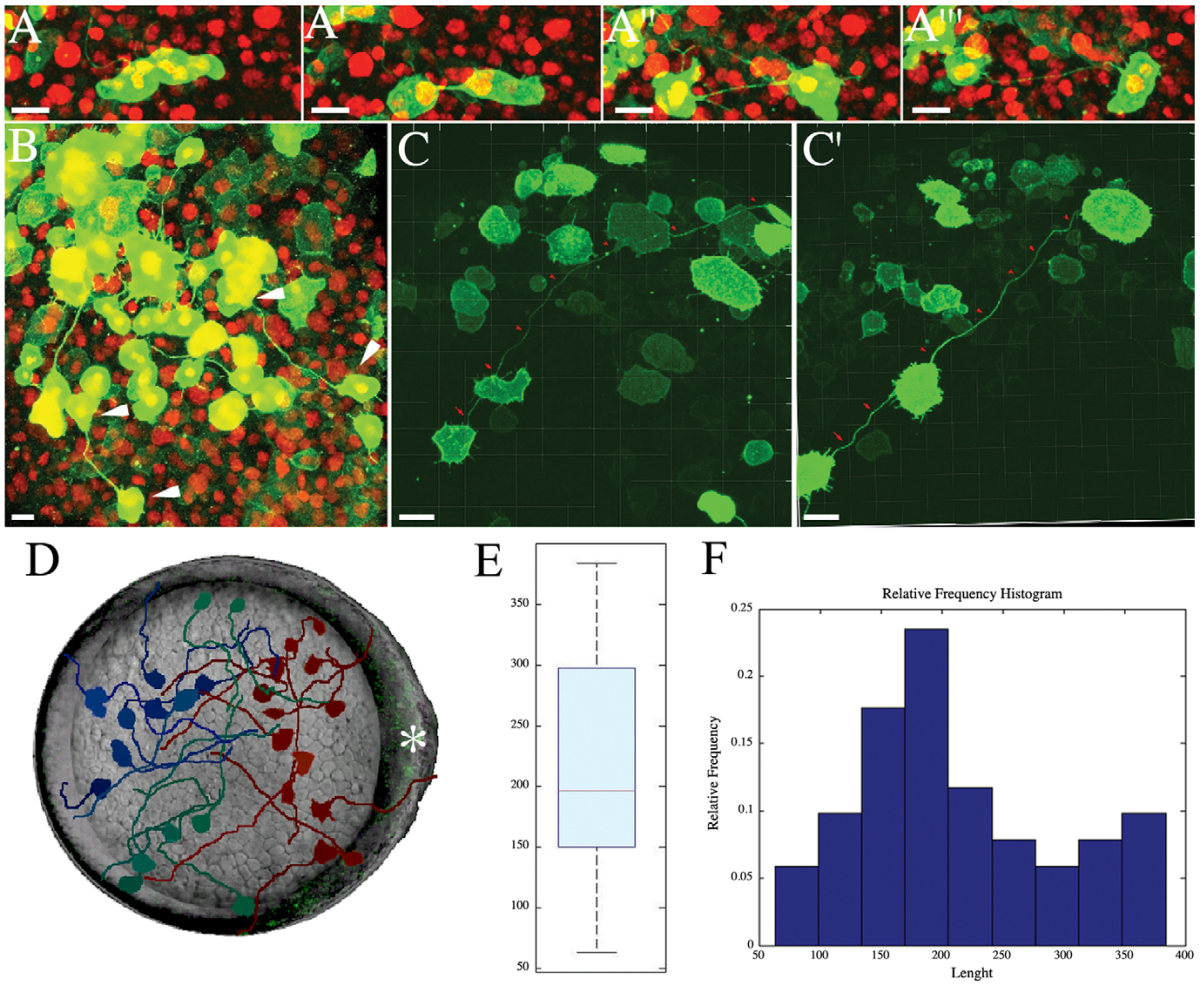
Intercellular Bridge Formation and Embryo Distribution. (A-A”’) Image series from a confocal time-lapse of a dividing cell forming an intercellular bridge during the blastula stage. Nuclei are labeled with H2B-mCherry (in red) and mDendra2 (in green) highlights the cellular boundary (see also Movie S1). (B) Animal pole view of blastula zebrafish showing several pairs of interconnecting cells (white arrows). (C, C’’) Persistence of the intercellular bridge (red arrow) from mid-gastrula (C) to the end of gastrulation (C’) (see also Movie S2). (D) Schematic showing several intercellular bridges mapped in an ideal embryo, shown here in an animal pole view. Different colors show different portions of the embryos: (red) neural plate region; (dark blue) non-neural territory; (green) presumptive lateral plate. The subdivision into territories has been made aligning each embryo by the embryonic shield and the anterior neural border. The dorsal side of the idealized embryo is shown by the white asterisk (*). (E, F) Quantification of the length of the intercellular bridges at midgastrula. (E) The boxplot shows the median length of intercellular bridges (red line). The first quartile (blue box) and the minimum and maximum value of the intercellular bridges (whiskers) are depicted for a total of n = 30 cells from 20 independent embryos. The average length of the intercellular bridges is 215 µm. (F) The histogram shows the same distribution represented in the boxplot. Scale bar (A-C’): 20 µm.

Three-dimensional imaging revealed that the cells linked by intercellular bridges resided within the epiblast layer; bridges were not observed linking cells in either the EVL or the hypoblast. A quantitative analysis of 51 intercellular bridges from 38 independent embryos at late gastrula stages showed that the protrusions averaged 215 µm in length (see Figure 2E). The imaging confirmed the presence of pseudopodia and other cellular extensions in the epiblast, but the length of such projections was a small fraction of the length of the intercellular bridges. Interestingly, previous reports of the cell morphologies during chick neurulation [11] and of deep cells in the medaka fish suggested the presence of intercellular bridges; in medaka a fraction of fixed and dissociated deep cells were found to be linked by short (up to 30 µm) intercellular extensions [12].

Time lapse studies of embryos show that the intercellular bridges form when a mitotic pair of daughter cells maintain a membrane tether, which is the lengthened as the cells move further apart and as neighboring cells subsequently intercalate between them (Figure 2A-A”’; see Movie S1). To document this association with mitoses, we captured confocal time-lapse movies of embryos ubiquitously expressing the chromatin marker Histone 2B fused to the red fluorescent protein mCherry (H2B-mCherry) [13] and watched as bridges were formed by the mDendra2-labelled cells. The intercellular bridges first appeared between dividing cells at the blastula stage; dividing cells only rarely gave rise to intercellular bridges at the gastrula stage. This formation mechanism, as a persistent mid-body from mitosis, differs from the active extension of the membrane observed for cytonemes in in the wing imaginal disc of *Drosophila melanogaster* [14] or for the protrusion of thin filopodia during sea urchin gastrulation [15].

The intercellular bridges persisted throughout gastrulation (Figure 2C-C’; see Movie S2), even as the cells executed morphogenetic motions such as radial intercalation. They were still visible as late as at the 10–13 somites stage (Figure S1B,C and Movie S3). While somewhat variable, about one of five cells were endowed with intercellular bridges during gastrulation. The bridges were not disrupted by subsequent mitotic activity of the cells (Movie S4). Cells equipped with intercellular bridges mitosed normally, suggesting that the bridges do not alter the oriented cell divisions present throughout gastrulation stage [16], [17]. Occasionally, we observed the fragmentation of an intercellular bridge, apparently when a migratory cell breaks through it (Movie S5). We have not captured the disruption of enough bridges to know if there are other mechanisms that act to reduce the number of intercellular bridges at later stages of development.

Quantitative analysis of the intercellular bridges during gastrulation showed that they lack any obvious orientation or preferential position. We superimposed bridges of 30 cell pairs from 20 different embryos at the mid-gastrula stage (Figure 2D), using the embryonic shield and the anterior neural boarder as morphological landmarks. The bridges do not respect boundaries in the fate map, bridging between neural and non-neural territories as well as within the same embryonic regions. We measured at mid-gastrula the angle between the putative antero-posterior axis in the neural plate and the shortest path between cells equipped with intercellular bridges (44 pairs from 23 independent embryos) and no obvious orientation was found (data not shown). This lack of alignment with the body plan and the intercellular bridges is expected because the axes of division in the blastula are not related to the later body plan or organ fate maps [1], [2].

Further characterization of intercellular bridges shows that there are minor variations in diameter (up to 1 µm) and significant variations in length, reaching lengths up to 350 µm (average span of 215 µm; Figure 2). The bridges are extraordinary in length when put in context with the individual cell diameters of 20–25 µm. The total surface area of a typical epiblast cell soma is around 1200 µm^2^, almost identical to the surface area of the longest intercellular bridge of 1 µm diameter (1100 µm^2^). Thus, the surface area of these membrane bridges increases the amount of cell surface of the linked cells, and the total present in the embryo.

The lumen of each intercellular bridge appears to be filled with cytoskeletal components and devoid of bulk cytoplasm. To sample the cytoskeleton present inside intercellular bridges we mosaically labeled cells within the embryos with membrane-targeted mCherry together with EGFP-β-Actin or EGFP-α-Tubulin. The images revealed that β-Actin was present at a low level for most if not all of the length of the intercellular bridges; α-Tubulin was detected only in the proximal portion of the intercellular bridges (Figures S1 and data not shown). In extremely long intercellular bridges, the contents seemed to thin in the middle, with the central part of the structure almost devoid of cytoskeletal components. The presence of cytoskeletal proteins within the intercellular bridges suggests they could be used to exert tension, and potentially acts as a cellular mechanical mediator.

**Figure 3:**
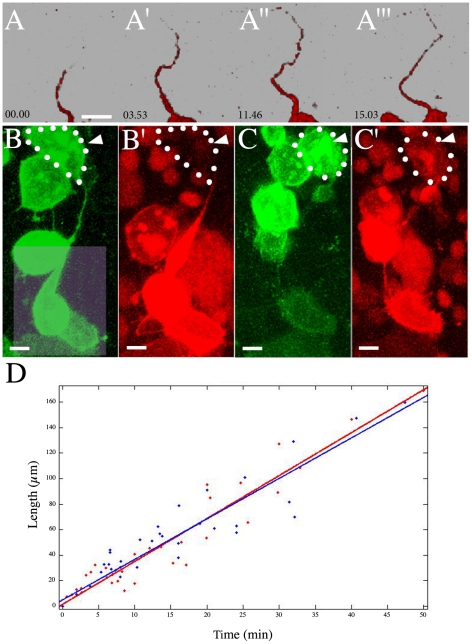
Membrane Dynamics of Intercellular Bridges. (A-A”’) Time-lapse rendering of photoconverted mDendra2 diffusing along the intercellular bridges (see also: Movie S4 and S6). (B-C’) Membrane transfer of photoconverted protein in embryos labeled with mDendra2. We photoconverted mDendra2 with a 405 nm light [7], which shifts the emission and excitation spectra. (B, B’) Non-converted mDendra2 positive cells are in green (B), and photoconverted cells are in red, with H2B-mCherry highlighting the nuclei (B’). The light square in (B) shows the region of photoconversion. (C-C’) The same cells are connected *via* an intercellular bridge. The pool of photoconverted mDendra2 reaches the cell at the opposite end of the intercellular bridge, indicated by the white arrowhead and white dotted outline. (D) Quantification of the dynamics — length as function of time — of a pool of photoconverted mDendra2 along the intercellular bridge (time in minute and length in µm). Scattered red and blue dots represent the raw data for mDendra2 and CD8-Dendra2, respectively. The curve fitting was performed using CurveFittingTool in MATLAB, and each curve has an R2 value of at least 0.9. Scale bar (A-C’): 10 µm.

To verify that the intercellular bridges are continuous between pairs of cells we photoconverted mDendra2 in one cell body and followed the motions of the photoconverted pool towards the other cell (Figure 3 and Movie S6). In all cases, the mDendra2 moved along the length of the intercellular bridge, eventually reaching the plasma membrane of the sister cell (Figure 3B-C'). The photoconversion of mDendra permitted us to measure the time required for a tagged protein to move along the length of the intercellular bridge. The movement seems faster than expected by diffusion alone, reaching distances of 100 µm in about 30 minutes (3.4 µm/min,). This fast rate is not particular to mDendra2; Dendra2 anchored via CD8 (CD8-Dendra2) [9], [10] was found to move at rate of 3.3 µm/min. The movement of the membrane-linked Dendra2 probes seems linear with respect to time, suggesting that it is due to some active process rather than by diffusion alone (Figure 3D). This rate of transfer along the intercellular bridges is sufficiently fast to permit a role in cell-cell communication during gastrulation. In cultured mammalian cells, tunneling nanotubes have been shown to be capable of organelle transfer [18], retroviral infection [19] and immune surface receptor transfer [20]. The motion of membrane-linked Dendra2 along the intercellular bridges seen here offers up the intriguing possibility that the intercellular bridges perform similar biological functions in zebrafish embryogenesis.

### Conclusion

During zebrafish gastrulation, a significant fraction of epiblast cells are endowed with intercellular bridges that extend to connect epiblast cells as far away as 350 µm. These intercellular bridges are formed by the maintenance of a cytoskeleton-filled membrane tether between pairs of dividing cells. The bridges persist for several hours. The rate of transfer of integral (CD8-Dendra2) and peripheral (mDendra2) membrane proteins along the intercellular bridges is fast enough to mediate cell-cell communication during gastrulation and neurulation. The formation, persistence, and material transfer along the intercellular bridges provide an unexpected pathway for the transfer of materials between cells in the zebrafish embryo. Their presence has important implications for not only membrane homeostasis, but also on the mechanical and signaling processes in embryonic patterning and development.

## Materials and Methods

### Zebrafish

Raising, maintaining, injection and spawning of wild-type AB zebrafish in house colony were performed as previously described [21].

### DNA Constructs

To generate embryos with high expression levels distributed in a mosaic subset of cells we used Tol2-mediated transient expression [8]. Briefly, the synthetic transposase mRNA and the transposon donor plasmid pMTB, which contains a β-Actin promoter flanked by minimal Tol2 *cis*-sequences necessary for transposition[22], [23], were co-injected into the zebrafish fertilized eggs. The Tol2 construct is excised from the donor vector and eventually integrated into the genome of somatic cells. To target the photoconvertible protein Dendra2 [7], the red fluorescent protein mCherry [13] to the plasma membrane, we used as a lipid anchor 2 repeats of a myristoylated and palmitoylated NH2-terminal MGCIKSKRKDNLNDDE signal sequence from Lyn kinase as previously described [9], [24]. The fusion constructs were generated by polymerase chain reaction primer extension, and further subcloned with ClaI and BamHI as the cloning sites into the transposon donor vector pMTB. Mouse CD8-Dendra2, a fusion protein between transmembrane mouse lymphocyte marker CD8 and Dendra2, was generated by subcloning a PCR product encoding the mouse CD8 (*mCD8*) gene [10] into the pCS2+Dendra2 vector with BamHI and XmaI as the cloning sites, generating a new ORF with Dendra2 fused in frame to the 3′ of mCD8. Finally, mCD8-Dendra2 was sub-cloned into the transposon donor vector pMTB with BamHI and SnaBI as the cloning sites. EGFP-α-Tubulin and EGFP-β-Actin were generated by subcloning both PCR products from the pEGFP-α-Tubulin vector and the pEGFP-β-Actin vector (Clontech), respectively, into the transposon donor vector pMTB vector with ClaI and SnaBI as the cloning sites. The H2B protein was tagged with mCherry [13] at its carboxyl terminus as previously described [25]. The H2B-mCherry gene were then sub-cloned into the transposon donor vector pMTB.

### Imaging, Dendra2 Photoconversion and Quantification

Injected Embryos were raised at 28°C in 30% Danieau solution [26] and developed indistinguishably from un-injected counterparts. Prior to imaging, specimens were oriented and placed in 1% low melting point agarose. Live images were obtained with a LD C-APO 40x/1.1 water objective on a Zeiss LSM 5 Exciter microscope setup. Photoconversion of Dendra2 was achieved by a 40 second exposure to intense 405 nm laser light. Images were processed using Adobe Photoshop CS3 (Adobe Systems), and analyzed using Imaris (Bitplane AG). The MATLAB (The MathWorks, Inc.) curve fitting toolbox was used to calculate the behavior of photoconverted Dendra moving along the intercellular bridges.

## Supporting Information

Figure S1 **Intercellular Bridge Properties.** (A) Animal pole view of a mosaic zebrafish embryo expressing Dendra2 at the onset of gastrulation. Note that intercellular bridges cannot be visualised even under intense 488 nm illumination of the cytoplasmic fluorescent protein. (B, C) Intercellular bridges visualised at tailbud stage (B) and 3 somites stage (C). (D–F) Intercellular bridge connecting two cells (white arrowheads) labelled with membrane targeted mCherry (D), EGFP-β-Actin (E), and overlay (F). Note that EGFP-β-Actin is partially present inside the intercellular bridge. Scale bar: 20 µm.
(TIF)

Movie S1 **Intercellular Bridge Formation (related to** **Figure 2** **).** Time-lapse imaging capturing the formation of an intercellular bridge after cell division at late blastula stage. Nuclei are labelled with H2B-mCherry (red) and cellular boundaries are labelled with mDendra2 (green). Scale bar 25: µm.
(AVI)

Movie S2 **Intercellular Bridge Persistence During Gastrulation (related to** **Figure 2** **).** Time-lapse imaging showing the stability of an intercellular bridge (red arrowheads) labelled with mDendra2 (green) from mid-gastrula to the end of gastrulation. Scale of the grid: 25 µm.
(AVI)

Movie S3 **Intercellular Bridge After Gastrulation (related to** **Figure 2** **).** Time-lapse imaging showing the presence of intercellular bridge labelled with mDendra2 (green) from 90% epiboly to 10 somites stage. Scale bar: 50 µm.
(AVI)

Movie S4 **Intercellular Bridges and Cell Division During Gastrulation (related to** **Figure 2** **).** Time-lapse imaging showing a cell equipped with an intercellular bridge labelled with photoconverted mDendra2 (red) that undergoes oriented cell division. Scale bar: 25 µm.
(AVI)

Movie S5 **Intercellular Bridge Fragmentation (related to** **Figure 2** **).** Time-lapse imaging showing the breakdown of an intercellular bridge labelled with mDendra2 (green; red arrowheads) by a migratory cell (white arrowhead). Scale bar 25 µm.
(AVI)

Movie S6 **Dynamic Behaviour Along Intercellular Bridges (related to** **Figure 3** **).** Representative image sequences of the displacement of photoconverted mDendra2 (red) along the plasma membrane of an intercellular bridge. Scale bar: 25 µm.
(AVI)
