# Supplementary figures and images for: Intercellular Bridges in Vertebrate Gastrulation
Source: PLoS One. 2011 May 25;6(5):e20230. doi: 10.1371/journal.pone.0020230 (PMC3102083; doi:10.1371/journal.pone.0020230)

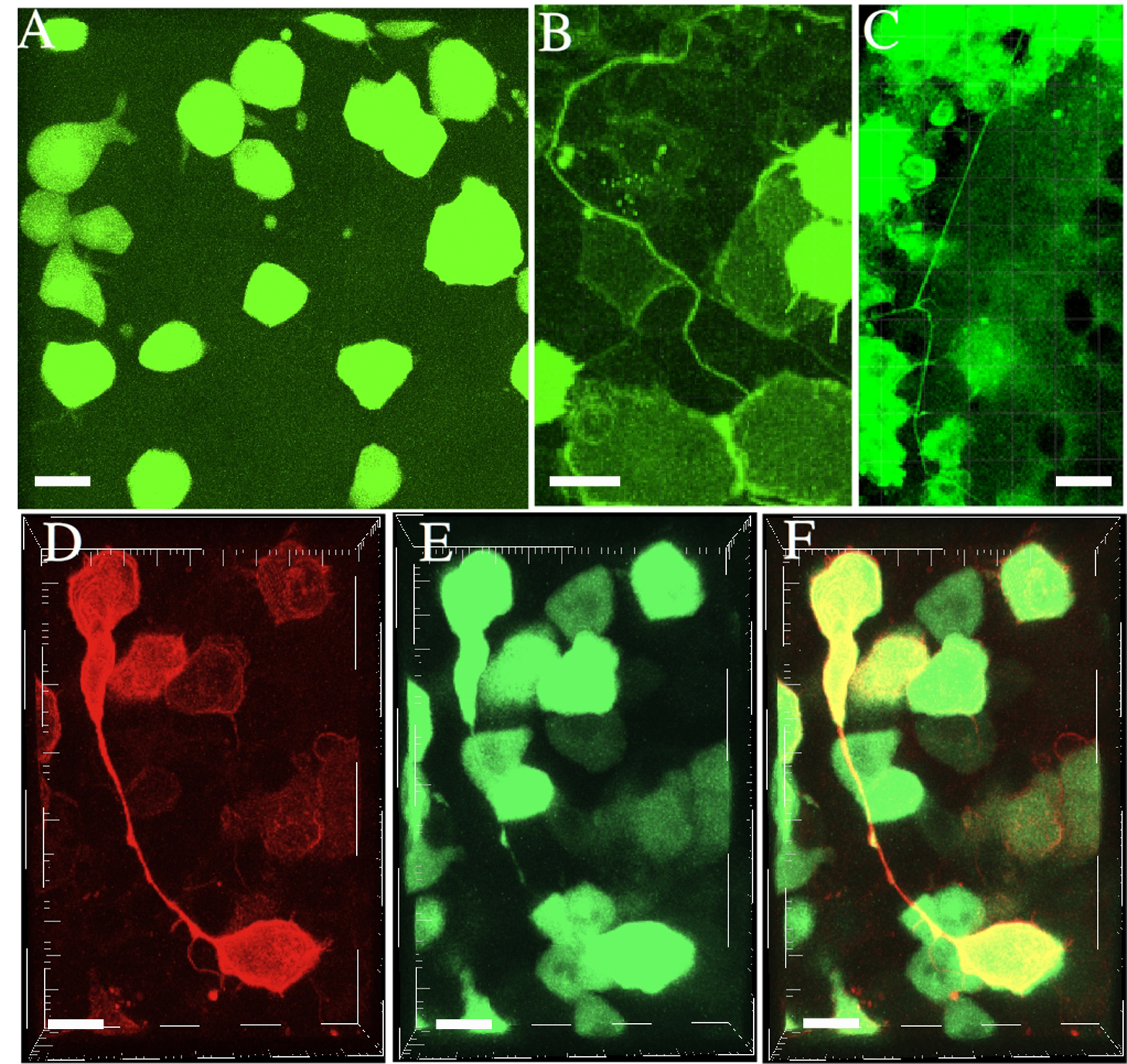

Supplement: Figure S1 — Intercellular Bridge Properties. (A) Animal pole view of a mosaic zebrafish embryo expressing Dendra2 at the onset of gastrulation. Note that intercellular bridges cannot be visualised even under intense 488 nm illumination of the cytoplasmic fluorescent protein. (B, C) Intercellular bridges visualised at tailbud stage (B) and 3 somites stage (C). (D–F) Intercellular bridge connecting two cells (white arrowheads) labelled with membrane targeted mCherry (D), EGFP-β-Actin (E), and overlay (F). Note that EGFP-β-Actin is partially present inside the intercellular bridge. Scale bar: 20 µm. (TIF) [file pone.0020230.s001.tif]
